# The Psychological Impact of Prophylactic Total Gastrectomy in Patients Who Are High Risk for Hereditary Diffuse Gastric Cancer: A Review of the Literature

**DOI:** 10.7759/cureus.84751

**Published:** 2025-05-24

**Authors:** Muhammad Y Hayat, Usman Yakubu, Jayan Jayasinghe, Bijendra Patel

**Affiliations:** 1 Trauma and Orthopaedics, University Hospital Southampton NHS Foundation Trust, Southampton, GBR; 2 Infectious Disease, The Royal Oldham Hospital, Manchester, GBR; 3 Colorectal and General Surgery, Barts Health NHS Trust, London, GBR; 4 Surgical Science, Barts Cancer Institute, London, GBR

**Keywords:** cdh1, clinical outcome, familial gastric cancer, hereditary diffuse gastric cancer, prophylactic total gastrectomy, psychological impact

## Abstract

High-risk patients for hereditary diffuse gastric cancer (HDGC) are commonly offered a prophylactic total gastrectomy (PTG). This includes patients with a germline CDH1 or CTNNA1 mutation and occasionally patients with variants of unknown pathogenicity. Whilst the psychological impact (PI) of curative total gastrectomy (TG) for gastric cancer (GC) has been well documented in the literature, there is a lack of consensus on how PTG affects this group of patients. Hence, this literature review aims to elucidate what is known about the PI of PTG.

A narrative review of the literature was carried out using a comprehensive search strategy on Ovid Medline, EMBASE, PubMed, SCOPUS, and Web of Science. After full-text screening, 12 citations were included to investigate the PI of PTG. Domains were classified for themes relating to the PI of PTG.

Themes that emerged for the PI of PTG included effects on emotional functioning, anxiety, depression, social life, body image, work and daily life, relationship with food, regret, cancer-related anxiety, and effects on intimate relationships.

All domains identified for the PI of PTG are complexly interlinked with the long-term clinical outcomes of PTG. All patients suffer from long-term morbidity, and those who tolerate PTG well, with improving physical symptoms over time, have better psychological outcomes. Alternatively, patients who experience persistent and severe long-term consequences of PTG have poorer psychological outcomes.

PTG is an established form of risk reduction, and care should be taken to address the PI of this procedure.

## Introduction and background

Genetic causes of hereditary diffuse gastric cancer

The CDH1 gene functions as a tumour suppressor gene located on chromosome 16 [1], which is inherited in an autosomal dominant manner [2,3]. It is transcribed into the transmembrane adhesion molecule known as E-Cadherin [1]. α-E-catenin, which is transcribed by CTNNA1, is also associated with this adhesion molecule [4].

Pathogenic variants of CDH1 and CTNNA1 have been linked to gastric cancer (GC) and lobular breast cancer (LBC) [4-6]. CDH1 dysfunction has been most significantly associated with the development of hereditary diffuse gastric cancer (HDGC), with historical estimates of cumulative incidence by 80 years of age ranging from 37-70% in men and 25-83% in women [7,8]. A recent study of 213 families in the United States concluded the cumulative risk of advanced diffuse gastric cancer (DGC) by age 80 to be 10.3% in males and 6.5% in females [9]. The most recent consensus guidelines have concluded that the HDGC risk varies between families, and hence, family history should also be taken into account when estimating an individual carrier’s risk [10].

Families that fulfil the criteria for HDGC are sent for CDH1 and increasingly, CTNNA1 genetic testing [6,10]. Of those tested, roughly 40% show a pathogenic CDH1 mutation [11], and a minority are positive for CTNNA1 [10].

Patients who are positive for mutations in CDH1 and CTNNA1 are considered to be high-risk patients for HDGC [6,10]. Those testing negative for a pathogenic gene variant despite a confirmed family history of DGC or LBC in first or second-degree relatives are termed to have a “HDGC-like” syndrome.

Management of high-risk patients

If a patient tests positive for known pathogenic variations of CDH1 and has a family history of DGC, they are offered a prophylactic total gastrectomy (PTG) [6,10]. Endoscopic surveillance with biopsy may be recommended if they have significant comorbidity, old age (>70 years) or if a patient wishes not to undergo a PTG [6,10].

The reason that a PTG is offered to high-risk patients with known cases of DGC in the family is because symptoms of early HDGC are vague, and specific symptoms begin to present in advanced stages of the disease [2]. This, compounded with the limitations of macroscopic detection in endoscopic surveillance [2], has led to PTG being offered in otherwise healthy patients.

The most recent guidance given by the International Gastric Cancer Linkage Consortium [10] is the first ever to advise follow-up for psychological effects of PTG [10]. It highlights that psychological interventions and outcomes for this group of patients are an emerging area of research [10].

In the literature, it is consistently assumed that the psychological impact (PI) is the same in those who undergo PTG compared with patients who undergo total gastrectomy (TG) for gastric cancer (GC) despite many differences in each of these populations (including younger age, lower number of comorbidities and not undergoing adjuvant chemotherapy). 

Further consensus and documentation of this data in the literature would allow for greater understanding of the PI in this population.

To date, no reviews exist on the PI of PTG patients who are high risk for HDGC.

Aims and objectives

What is present in the literature on the PI of PTG in patients who are high risk for HDGC? Objectives of the PI to be explored include the effect of PTG on emotional function, anxiety, and depression. The effect of PTG on Social Life and the effect of PTG on self-perceived body image.

## Review

Methods

This is a narrative review of retrospective and prospective cohort studies, as well as case series and cross-sectional studies from published and unpublished literature, collating patients who are high risk for developing HDGC and have undergone a PTG.

A narrative review design was utilised due to the paucity of data in the literature on HDGC. Furthermore, published literature tends to have small cohort sizes due to the clinical rarity of PTGs. Hence, this design allows for thematic analysis.

PI is defined as the impacts of PTG which affect the mental health and emotional state of patients.

**Figure 1:**
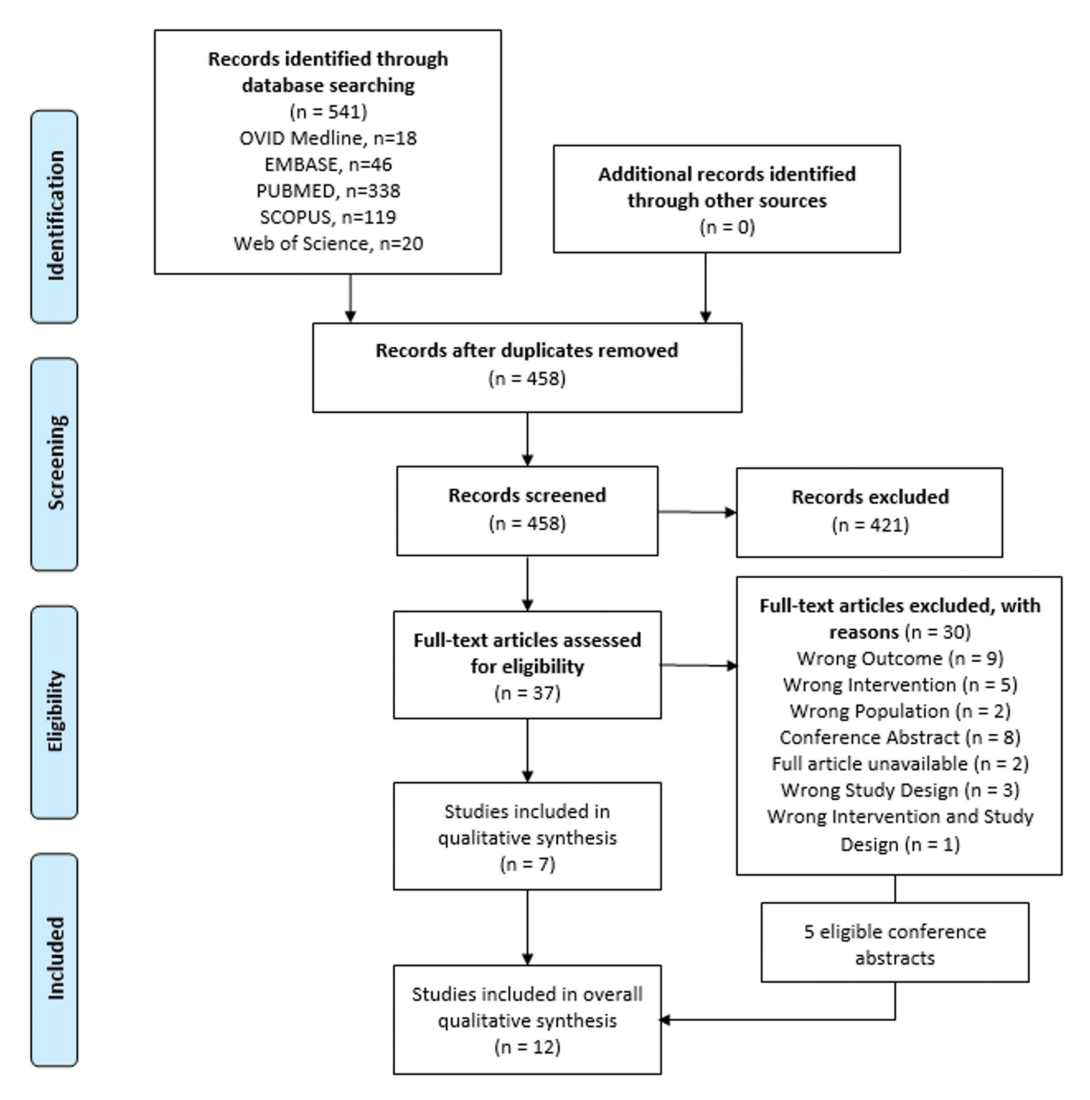
Study flow diagram for the psychological impact of PTG PTG - prophylactic total gastrectom

Modified PRISMA guidelines [12] were used to create a study flow diagram (Figure 1).

A comprehensive literature search was carried out on OVID Medline, EMBASE, PUBMED, SCOPUS and Web of Science using the search strategy in Appendix 1. 

After collating citations, duplicates were removed using EndNote Referencing software [13] and manual screening too. Seven citations were initially included for qualitative synthesis. Conference abstracts were further screened as it was deemed that they would make a valuable addition to this literature review. Three abstracts were further excluded. This is because they summarised the same findings as other included citations, but presented them at different conferences. Five conference abstracts were eligible for inclusion in qualitative synthesis, making a total of 12 included citations. Study characteristics can be seen in Table 1.

**Table 1:** Study characteristics table of included studies

Study title	Study design	Year of publication	Lead author
The Psychosocial Impact of Undergoing Prophylactic Total Gastrectomy (PTG) to Manage the Risk of Hereditary Diffuse Gastric Cancer (HDGC)	Retrospective cohort study (qualitative – grounded theory)	2016	Hallowell et al. [14]
The Impact of Prophylactic Total Gastrectomy on Health-Related Quality of Life	Prospective cohort study	2014	Worster et al. [15]
Prophylactic Total Gastrectomy: A Prospective Cohort Study of Long-Term Impact on Quality of Life	Prospective cohort study	2016	Muir et al. [16]
Hereditary diffuse gastric cancer: cancer risk and the personal cost of preventive surgery	Retrospective cohort study	2019	Kaurah et al. [17]
Paving the Way: A Grounded Theory of Discovery and Decision Making for Individuals with the CDH1 Marker	Retrospective cohort study (qualitative – grounded theory)	2020	Hersperger et al. [18]
Therapeutic and prophylactic gastrectomy in a family with hereditary diffuse gastric cancer secondary to a CDH1 mutation: a case series	Case series	2018	Gjyshi et al. [19]
“How do you live without a stomach?”: A multiple case study examination of total gastrectomy for palliation or prophylaxis	Case series	2011	Garland et al. [20]
Decision making and the psychosocial impact of prophylactic gastrectomy	Cross-sectional study (conference abstract)	2011	Kluijt et al. [21]
Prophylactic gastrectomy in CDH1-mutation carriers: Psychosocial, physical, and nutritional effect compared with curative gastrectomy for gastric cancer.	Retrospective cohort study (conference abstract)	2011	Cats et al. [22]
“Life changing” the lived experience of risk reducing gastrectomy in people at risk of hereditary diffuse gastric cancer	Case series (conference abstract)	2013	Young et al. [23]
Patient reported outcomes (PROs) after prophylactic laparoscopic gastrectomy in five siblings with germline mutation of the E-cadherin gene	Prospective cohort study (conference abstract)	2011	Mayrbäurl et al. [24]
Multidisciplinary care for CDH1 carriers opting for prophylactic gastrectomy	Cross-sectional study (conference abstract)	2013	Bleiker et al. [25]

The database search and screening of studies for inclusion were completed in July 2021. However, two additional studies published thereafter have been identified. Their findings are clearly discussed and contextualised in the discussion section to provide a comprehensive and up-to-date overview of this research area.

Minimising Bias

There was variation amongst included studies for the definition of PTG. All patients who had morphologically normal stomachs upon endoscopy and were asymptomatic pre-surgery, despite pre-operative biopsy and post-operative pathological staging, were included, as the intention of this procedure was for prophylactic purposes.

Results

Demographic Results

A summary table of the demographic results is seen in Table 2.

**Table 2:** Summary table of demographic results of included citations PTG - prophylactic total gastrectomy Raw data in Table 5 (Appendix)

166 PTG patients: (9/12 citations)		
Biological Sex	Male	58/166 (34.94%)
	Female	108/166 (65.06%)
	Ratio (M:F)	01:01.9
206 PTG patients: (11/12 citations)		
Mutation status	CDH1 positive	202/206 (98.06%)
	Mutation negative	4/206 (1.94%)
114 PTG patients: (6/12 citations)		
Age of patients	Mean (SD)	35.64 (10.58) years
120 PTG patients: (8/12 citations)		
Age of patients	Minimum - Maximum	16 - 68 years

In the 12 included citations, there were a total of 230 participants. Of these participants, 213 (92.6%) underwent a PTG.

Data on biological sex of participants undergoing PTG was available for 166 PTG patients. Fifty-eight patients (34.94%) were male and 108 (65.06%) were female. This represents a ratio of one male for every 1.86 females.

Genetic mutation status was available for 206 patients who underwent PTG, and 202 (98.06%) were positive for the CDH1 gene, and four (1.94%) were negative.

The mean (standard deviation) age was calculated from six citations, which included 114 PTG patients. Mean age was calculated based on mean and median values. The mean age (SD) of PTG patients was 35.64 (10.58) years.

The minimum-maximum ages of PTG patients were calculated from data on 120 patients, and they ranged from 16 to 68 years old.

Intervention Results

A summary table of the intervention results is seen in Table 3.

**Table 3:** Summary table of intervention results of included citations PTG - prophylactic total gastrectomy, SRC - signet ring cells Raw data in Table 6 (Appendix)

42 PTG patients: (3/12 citations)		
Pre-operative biopsy	Positive	16/42 (38.10%)
	Negative	26/42 (61.90%)
114 specimens (6/12 citations)		
Pathological report	Total positive for SRC	88/114 (77.19%)
	SRC foci	80/114 (70.18%)
	pT1a	7/114 (6.14%)
	pT3a	1/114 (0.88%)
	Negative	26/114 (22.81%)
118 PTG patients (7/12 citations)		
Follow-up Length	Mean	26.2 months
140 PTG patients (5/12 Citations)		
Follow-up length	Minimum - maximum	6 - 108 months

Data on pre-operative gastric biopsy status were available in three included citations. Of 42 patients who underwent PTG, 16 (38.10%) had positive pre-surgical biopsies, whilst 26 (61.90%) had negative pre-surgical biopsies.

Data on pathological reports post-PTG was calculated from six citations. Of 114 pathological specimens, 88 (77.19%) had positive findings for signet ring cells (SRCs). Eighty were positive for SRC foci (70.18%), seven were positive for T1a foci (6.14%), and one patient was positive for a pT3a focus (0.88%). Twenty-six (22.81%) PTGs out of 114 were truly prophylactic with no pathological evidence of SRCs.

The mean length of follow-up was calculated from the mean and median values of seven included studies of 118 PTG patients. Mean follow-up was 26.2 months. The minimum and maximum follow-up length was calculated from 140 PTG patients (over five citations) and was 0.5-9 years.

Thematic Analysis Results

**Figure 2:**
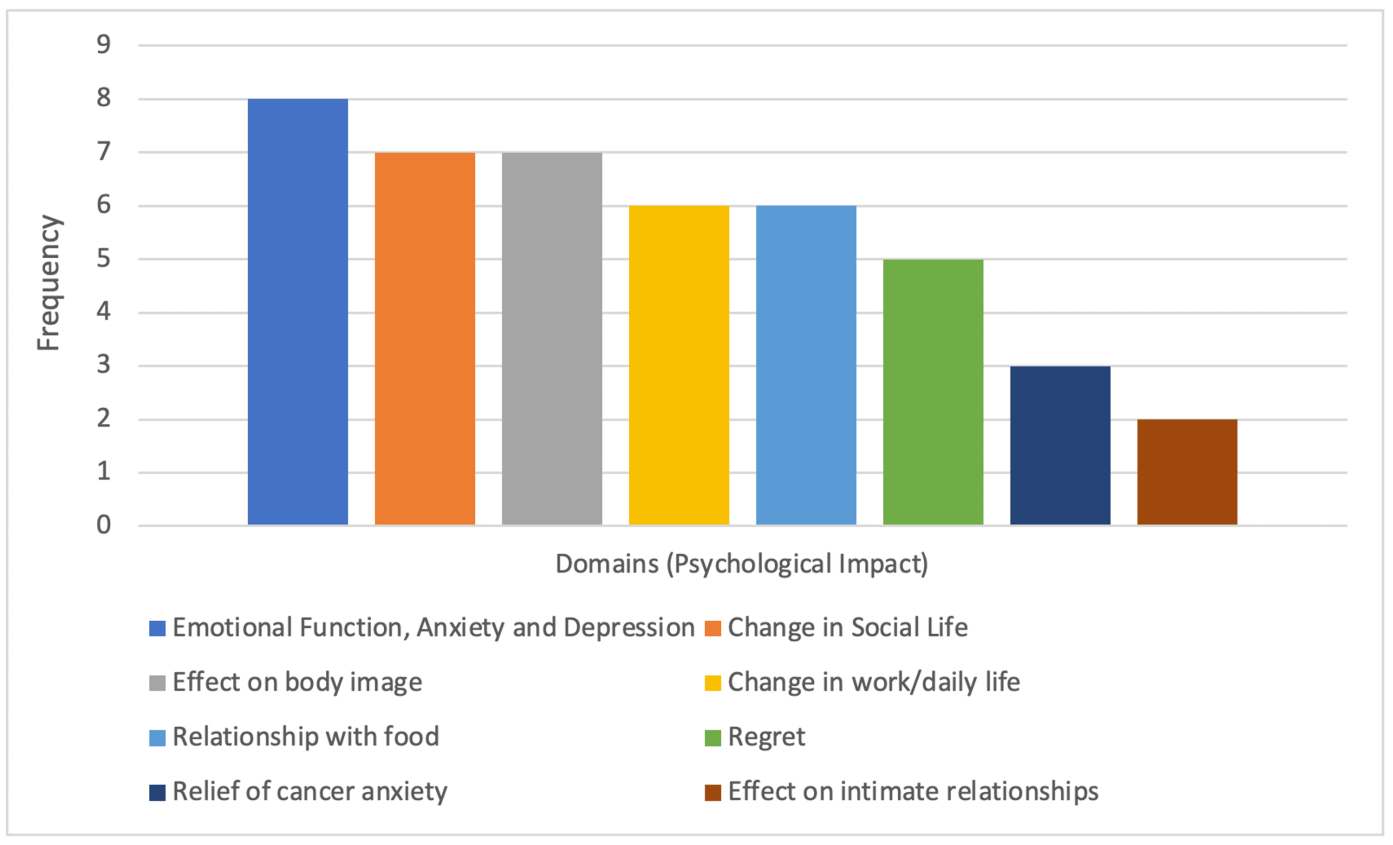
Frequency of domain establishment in included citations Analysis of the quality of domain establishment is displayed in Table 7 (Appendix)

Thematic analysis of the included studies was carried out and the frequency of domain establishment in included studies is depicted (Figure 2).

Each included study was analysed further based on how well each domain had been established. This is displayed in Table 7 (Appendix).

Discussion

Changes in Emotional Functioning, Anxiety, and Depression

Patients described their experience with PTG as mentally challenging in the short and long term [23]. Mayrbaurl et al. [24] reported that the greatest functional impairment was in emotional functioning from pre-PTG baseline [24]. 

Mental health and emotional functioning were shown to drop significantly a month post-surgery but subsequently recovered alongside physical functioning [15]. A strong correlation was found between mental well-being and physical functioning [15]. Kaurah et al. [17] found that 54% of PTG patients experienced anxiety, and 67% experienced depression to a greater degree than average population values [17]. Patients in their cohort that experienced severe depression or anxiety were all diagnosed with a previous mental health disorder [17], and hence, it was inferred by multiple citations that PTG patients experience mild symptoms of anxiety and depression [16,17].

Building on the thematic analysis of the included citations, a retrospective cohort study by Gallanis et al. [26], identified after screening, further elaborates on post-operative depression. They noted that 15 patients self-reported a pre-operative diagnosis of general anxiety disorder, depression or bipolar disorder with a further five patients of their cohort developing a new diagnosis of depression or anxiety post-operatively [26]. One patient developed alcohol dependence, which required rehabilitation post-operatively [26].

Anxiety was shown to be a significant predictor variable for quality of life (QOL) post-PTG [17]. Muir et al. [16] demonstrated that at every post-operative time point post-PTG, patients reported feeling anxious about their health status and had deficits in emotional functioning [16]. Many patients exhibited anxiety in the post-operative period in relation to muscle loss, social and economic changes [14,19].

Cats et al. [22] found that long-term anxiety and depression did not significantly vary between GC patients and PTG patients [22], which indicates recovery over time. Worster et al. [15] emphasised that the course of recovery of mental health post-PTG is not comparable to those who have GC, as anxiety and depression would decrease post curative-surgery [15] with the hope of being cured, and this has been established in the literature [27].

One patient who had a strong support network reported feeling optimism about their future and displayed increased emotional maturity after long-term recovery [20]. Their experience of PTG combined with the death of a close family member allowed them to empathise more with others and become proactive in resuming their life the way it was pre-PTG [20]. Unfortunately, we do not know which patients received counselling and how targeted counselling and pharmacological intervention influenced levels of anxiety and depression.

Changes in Social Life

Social function was found to be a significant predictor variable for overall QOL [17].

Gastrointestinal symptoms post-PTG was a common sub-theme which caused disturbances in social life. Events that revolved around foods, such as meeting for lunch, were avoided by patients as there was a possibility of them “being sick” [14]. Other patients reported that diarrhoea had prevented them from going out and socialising unless they knew that toilets were available [14]. Patients with gastrointestinal symptoms affecting their social life did not report any solutions they found and tended to avoid these situations instead.

Fatigue also influenced social life. Patients reported that they limited social activities to factor in resting [14], and the unpredictable nature of fatigue interfered with social activities [14]. 

Often, a combination of factors led to a decline in social life. Patients found that fatigue, change in diet, inability to drink alcohol and poor finances due to convalescence hindered their social activities [14]. Worryingly, some patients reported losing friends as they had become a “completely different person” [14].

Patients reported a social role change by becoming an educator about their gastrectomy [14,20]. Public unfamiliarity with PTGs meant that patients found themselves having to increase awareness of this condition, and it was taxing on them [14,20].

Changes in the use of social media were reported too [18]. This was to inform family members to get tested for the CDH1 gene and for peer-to-peer emotional support and counselling through online support groups [18]. Hersperger et al. found this to be critical for adjusting to a new normal as it helped patients to understand what to expect post-PTG [18].

Muir et al. [16] found that social functioning immediately decreased post-PTG but climbed to baseline (with no deficits) by 12 months post-operatively [16]. Social functioning then decreased again by 24 months post-PTG [16]. Patients at this time point reported continuation of gastrointestinal symptoms and financial difficulties [16], which may have been responsible for a decline in social functioning. This phenomenon was also reported by Worster et al. [15], where social functioning was recorded at its lowest a month post-PTG, increased towards baseline by a year and declined again by 24 months. 

Lack of recovery of social function in young patients at a socially pivotal time in life may have long-term consequences, as they are not able to fit in with their peers. Hallowell et al. [14] reported that adaptations in social life post-PTG negatively impacted patient self-identity [14]. Literature on social function post TG for GC revealed a long-term recovery in social function to a level equal to or greater than their previous baseline [28,29]. This may be because there are more support groups for patients with GC compared to those who undertake a PTG. Patients who have a TG for GC are usually older [22], hence, social pressures may differ, and patients may find curative TG more worthwhile than a PTG.

Variability in experience post-PTG influenced social function, too. Young patients (under 30) who recovered to what felt ‘normal’ reported very little effect on lifestyle whereas young patients who suffered from long-term gastrointestinal morbidity reported effects on social life years down the line [14]. 

Changes in Body Image and Self-Identity

Impact of PTG on men’s body image was a recurring sub-theme. Men often expressed anxiety regarding muscle and strength loss post-PTG [14,19,20]. This negatively impacted feelings of masculinity, and in combination with fatigue, this meant that some manual workers were no longer able to continue their lifelong careers, which negatively impacted self-identity [14]. Participants reported fixation on looking at their body in the mirror with sadness regarding muscle loss [18].

Garland et al. [20] commented on this theme by postulating that women may not have found weight loss to be as problematic to their self-perceived body image as men [20].

Worster et al. [15] reported that 44% of patients had persistent changes in self-perceived body image, and this was identified as not fully recovering even 24 months post-PTG [15]. Kaurah et al. [17] found that changes in body image did not significantly impact overall QOL [17], and Muir et al. [16] reported that the body image of patients was relatively unaffected over time [16]. However, females vastly outnumbered males in their samples, and they did not account for time elapsed since surgery [16,17].

Despite this, a group of patients in this study cited weight loss as their biggest concern [16], and some patients experienced worsening in self-perceived body image after the 12-month mark [16].

Studies on TG patients have found that significant muscle loss could be prevented by strict nutritional follow-up and adequate caloric intake [30]. This was reported by Gjyshi et al. [19] where a PTG patient experienced anxiety regarding muscle loss, and with strict nutritional support, they were allowed to resume arm wrestling which was their hobby [19].

One female patient reported that PTG positively impacted their body image, as they were proud of their surgical scars [14]. Despite this, the overwhelming majority (especially men) were negatively impacted.

Changes in Work Life and Daily Activities

There was variation in resumption of daily activities post-PTG, with some patients being back to work within six months of recovery and others being unable to return to work [14]. Patients who were unable to return to work tended to have prolonged morbidity associated with their PTG, such as dumping syndrome, weight loss and fatigue [14,21].

Gallanis et al. [26] reaffirm this finding. They noted that 23.5% of their cohort reported a change in employment post PTG, which was often attributed to persistent post-operative symptoms, namely gastrointestinal symptoms, fatigue and nausea [26].

According to Kluijt et al. [21], severe fatigue led to 40% of patients having severe impairments in their daily activities a year post-PTG [21]. Another citation showed that severe fatigue post-PTG and severe impairments in daily activities (including hobbies and work) were exhibited in up to 48% of patients [22].

Patients suffering from severe fatigue post-PTG due to inadequate caloric intake felt that it was hard to carry out basic daily activities [14]. Other patients reported similar experiences, and one patient stated that going for a walk would confine them to bed for two days [14]. Those suffering from chronic fatigue had to drastically reduce the number of hours they worked [14]. This led to financial impacts, which also had ramifications on social life and daily activities [14]. Patients whose work involved manual labour were affected by this the most, often becoming unemployed [14], but sedentary office workers were also affected [14]. 

Poor role functioning in these patients was shown to be a significant predictor of QOL [17]. Role functioning was primarily influenced by weakness, diarrhoea and fatigue [17].

Young patients felt as though taking time out of education in their early twenties to undergo PTG would have long-term financial consequences [14]. Despite this, Hallowell et al. [14] reported that patients of all age groups experienced financial difficulties, which was related to the inability to work full time due to chronic gastrointestinal symptoms or fatigue [14].

Patients who were unable to continue their hobbies due to difficulty with oral intake and underwent nutritional support were able to resume their hobbies over time [19].

Changes in Patient Relationship with Food

Young et al. [23] mentioned that the effects of PTG on patients’ relationships with food were “life changing” and that recovery was more complex than simply physical healing [23].

A recurring theme in relationships with food post-PTG was that there was no typical post-PTG diet [14,20]. Patients often reported going through a trial phase to find what worked best for them [14,20]. The uncertainty of a post-PTG diet was a source of anxiety for some patients [14], particularly a case of two siblings who had a PTG and ate “completely different” foods [14].

Taste loss post TG has been frequently reported in the literature [31] as well as aversion to certain foods, which may partially explain the variation in food tolerability post-PTG [17].

Cats et al. [22] showed that troublesome foods were often dairy, fried products and soft drinks [22]. These are usually “comfort foods” and this theme re-occurs in the literature [20]. Patients reported that they were unable to consume the comfort foods and portion sizes that they previously enjoyed [14,20].

Enjoyment of food declined post-PTG [14,20]. Meals and snacks had to be planned in order to avoid physical manifestations such as feeling lightheaded and shaky [18,20]. This was due to an inability to gauge fullness post-PTG [18,20]. The conscious act of forceful food consumption was a sub-theme that emerged where patients forced themselves to eat calorie dense food to remediate weight loss post-PTG [14,20]. All patients who reported this behaviour were male.

Discomfort associated with regimented food consumption was an emasculating experience [14], and patients often reported that it became easier to cope with this over time as they became used to it [14,18,20]. This was usually through trial and error, such as by making sure they chewed their food more than usual [20] or by modifying their diet to avoid foods that made them feel unwell [14].

Pain and eating restrictions improved over time [15], which may explain why dietary modifications became easier for patients.

Regret and Relief of Cancer-Related Anxiety

Kluijt et al. [21] found that 90% of patients who underwent PTG never regretted their decision, and those who declined a PTG experienced more anxiety regarding HDGC [21] and anxiety induced by endoscopic surveillance [20]. This was found to be a major benefit of having a PTG as it allowed for closure of their worries about developing cancer [14]. 

Patients undergoing PTG tended to weigh the chances of developing cancer and potential for longer survival, and stated that a change in their relationship with food was worth potentially living longer [20]. 

Patients with less post-surgical gastrointestinal symptoms regretted their PTG less than those with more symptoms [14]. Muir et al. [16] found that 50% of PTG patients expressed regret at two to four weeks post-PTG; however, this declined over time as physical symptoms improved [16]. Similarly, Kaurah et al. found that 88% of participants were satisfied with their PTG [17], but those who were dissatisfied experienced more physical symptoms upon follow-up [17].

A prospective natural history study by Gamble et al. [32] was identified after screening. Patients were administered a decision-regret survey post PTG [32]. Individuals with no pathological evidence of cancer and those who suffered from post-operative complications and morbidity were more likely to experience regret [16,32].

In the literature, women who had prophylactic bilateral mastectomies experienced greater regret when there were poorer cosmetic outcomes, chronic pain, infection and lymphoedema [33]. This reaffirms the notion that regret after prophylactic procedures is experienced more in those who display more physical symptoms.

By undergoing PTG, a young patient felt that they could move on and “start” their life [20]. This patient’s views were influenced by witnessing the traumatic event of their sibling passing away from metastatic DGC [20]. Hence for this patient, PTG gave them relief [20].

Patients with family members affected by DGC had strong preferences for PTG and reported less regret [20]. This may have been due to the perceived cancer risk [20]. Perceived cancer risks and witnessing a relative go through treatment for cancer have been established in the literature as influential over prophylactic surgical decisions [34].

Prophylactic surgery, especially prophylactic bilateral mastectomy, has been frequently reported in the literature to reduce chronic cancer-related anxiety and worry with positive psychosocial impacts [35].

Effects on intimate relationships

Cats et al. reported that 18-48% of PTG patients had severe impairment in personal and intimate relationships [22]. Gallanis et al [26] noted that two patients in their cohort had divorced post-operatively [26].

Some patients mentioned that their sexual function and expression were negatively affected by PTG to the point that it was almost non-existent [14]. A possible theory behind this is due to long-term caloric restriction post-PTG. Caloric deficits, even despite adequate nutrition, have been shown to cause a decrease in serum testosterone level [36].

In the post-surgical period, patients described that intimate partners and close family members became carers for them [14]. Patients recommended that the decision to proceed with a PTG should be sought when adequate consideration is given to the availability of post-operative support from partners and family members [14].

Having to watch loved ones suffer from undertaking a PTG or from DGC was emotionally taxing, and in some cases, influenced the decisions of patients too [14,20].

Limitations

All studies used convenience samples and had high selection bias due to the clinical rarity of PTG. This is another source of bias, which cannot be limited in this case.

Subgroup analysis based on psychological outcomes for patients undergoing open vs minimally invasive surgery was not possible. This limitation arose as 11 of the 12 included studies did not explicitly classify data on the route of access and how this distinction impacted the PI of PTG in their findings.

Literature recommendations

Further research is recommended on all domains identified to do with the PI of PTG. Specific Recommendations include the impact of psychological support and nutritional support in patients who have had a PTG, the effects of weight loss post-PTG on the body image of both male and female patients. The effect of social media on information giving and communication for support is recommended for further research.

Feelings of regret should be further analysed in patients with foci of SRCs as opposed to those with pathologically normal stomachs.

Clinical recommendations

Support groups for PTG patients do not currently exist in the UK. This is an area for future clinical recommendations. Patients who are struggling to adjust in the post-operative period should have graded intervention based on the severity of the impact of PTG on their mental and physical health.

## Conclusions

Changes in mental health following PTG are distinct from GC patients, and the PI of PTG is complexly interlinked with clinical outcomes.

All identified domains of psychological functioning correlate strongly with physical symptoms experienced after PTG. Every patient suffers from long-term morbidity, and for patients who tolerate PTG well, as physical symptoms improve over time, psychosocial symptoms improve too (after a period of initial adjustment). Conversely, patients who experience persistent long-term symptoms have poorer psychological, social, and economic outcomes and QOL.

PTG is an established form of risk reduction in this group of patients, and care should be taken on a case-by-case basis to address the physical and psychological symptoms experienced in the short and long-term post-operative period to allow for greater adjustment to a new normal.

## Appendices

**Table 4:** Search Terms and Alternative Terms Used in The Literature Search

	Search Term:	Alternative Term:	Alternative Term:	Alternative Term:	Alternative Term:	Alternative Term:	Alternative Term:	Alternative Term:	Alternative Term:
P	CDH1	CDH1 Proteins	Familial Gastric Cancer	Hereditary Diffuse Gastric Cancer					
I	Prophylactic Total Gastrectomy	Prophylactic Gastrectomy	Gastrectomy						
C	-	-	-	-	-	-	-	-	-
O	Psychological Impact	Stress	Psychological Distress	Psychological Trauma	Psycholog$ (truncated)	Psychosocial	Mental Health	Depression	Anxiety

**Table 5:** Raw Data on Demographic Results of Included Citations Summarised in Table 2

Study:	Participants:	Number with PTG	Males:Females	CDH1 Mutation Status:		Age of PTG patients at surgery:
				Positive:	Negative:	
Hallowell et al. [14]	27	27	13:14	24	3	Median = 36, Range: 19-64
Worster et al. [15]	32	32	15:17	32	0	median = 35, Range: 16-64
Kaurah et al. [17]	53	53	14:39	53	0	34<50y.o., 19>50y.o.
Gjyshi et al. [19]	3	2	1:1	2	0	Mean = 27.5, Range: 23-32
Muir et al. [16]	18	13	2:11	12	1	Median = 51, Range: 23-63
Hersperger et al. [18]	17	13	3:10	13	0	-
Garland et al. [20]	3	1	1:0	1	0	Mean = 18
Kluijt et al. [21] (abstract)	25	20	-	20	0	-
Cats et al. [22] (abstract)	20	20	9:11	20	0	Mean = 41, Range: 20-68
Young et al. [23] (abstract)	7	7	-	-	-	-
Mayrbäurl et al. [24] (abstract)	5	5	0:5	5	0	31-36 = range
Bleiker et al. [25] (abstract)	20	20	-	20	0	Mean = 41, Range: 20-63

**Table 6:** Raw Data for Intervention Results of Included Citations Summarised in Table 3

Study:	Pre-operative Biopsy:		Histology post PTG:	Follow up period/time since PTG:
	Positive:	Negative:		
Hallowell et al. [14]	13/27	14/27	-	Median = 3 years, Range: 0.5-9 years.
Worster et al. [15]	-	-	27/28 SRC foci (96.43% positive)	Median = 2 years
Kaurah et al. [17]	-	-	32/53 SRC foci (60.38% positive)	Range: <12 months - >60 months
Gjyshi et al. [19]	1/2	1/2	2/2 patients = pT1a, (100% positive)	-
Muir et al. [16]	2/13	11/13	8/13 SRC foci, 1/13 pT3a (69.23% positive)	Median = 24 months
Hersperger et al. [18]	-	-	13/13 SRC foci (100% positive)	-
Garland et al. [20]	-	-	-	Mean = 9 months
Kluijt et al. [21] (abstract)	-	-	-	Mean = 2.7 years, Range: 15-80 months
Cats et al. [22] (abstract)	-	-	-	Mean = 34 months, Range: 5-63 months
Young et al. [23] (abstract)	-	-	-	-
Mayrbäurl et al. [24] (abstract)	-	-	5/5 T1a foci (100% positive)	Mean = 2 years (for every patient)
Bleiker et al. [25] (abstract)	-	-	-	Range: 2 months – 5 years

**Table 7:** Domain Categorisation of Included Citations Summarised in Figure 2

	DOMAINS COVERED:							
Study:	Relationship with food/ Anxiety around Eating:	Change in social life:	Problems with work life/daily activities:	Relief of Cancer related anxiety:	Threats to identity/changes in body image:	Problems with intimate relationships:	Changes in Emotional Functioning, Anxiety and Depression	Regret:
Hallowell et al. [14]	Well established	Well established	Well established	Moderately established	Moderately established	Moderately established		Poorly Established
Worster et al. [15]		Moderately established			Moderately established		Well established	
Kaurah et al. [17]	Moderately established	Well Established	Moderately established		Moderately established		Well established	Poorly Established
Gjyshi et al. [19]			Poorly Established		Poorly Established		Poorly Established	
Muir et al. [16]		Moderately established			Well established		Well established	Well established
Hersperger et al. [18]	Moderately established	Moderately Established			Poorly Established			
Garland et al. [20]	Moderately Established	Moderately established		Moderately established	Moderately Established		Poorly established	Moderately established
Kluijt et al. [21](abstract)			Poorly Established	Poorly Established				Poorly established
Cats et al. [22](abstract)	Moderately Established		Poorly Established			Poorly Established	Poorly Established	
Young et al. [23](abstract)	Poorly Established	Poorly Established					Poorly Established	
Mayrbäurl et al. [24] (abstract)							Poorly Established	
Bleiker et al. [25](abstract)			Poorly Established					
